# A decrease in the age of respired carbon from the terrestrial biosphere and increase in the asymmetry of its distribution

**DOI:** 10.1098/rsta.2022.0200

**Published:** 2023-11-27

**Authors:** Carlos A. Sierra, Gregory R. Quetin, Holger Metzler, Markus Müller

**Affiliations:** ^1^ Department of Biogeochemical Processes, Max Planck Institute for Biogeochemistry, Jena 07745, Germany; ^2^ Department of Earth System Science, Stanford University, Stanford, CA 94305, USA; ^3^ Department of Geography, University of California, Santa Barbara, CA 93106, USA; ^4^ Department of Crop Production Ecology, Swedish University of Agricultural Sciences, Uppsala 75651, Sweden; ^5^ Center for Ecosystem Science and Society, Northern Arizona University, Flagstaff, AZ 86011, USA

**Keywords:** global carbon cycle, radiocarbon, terrestrial ecosystems, land surface models, Earth system dynamics, global biogeochemical cycles

## Abstract

We provide here a model-based estimate of the transit time of carbon through the terrestrial biosphere, since the time of carbon uptake through photosynthesis until its release through respiration. We explored the consequences of increasing productivity versus increasing respiration rates on the transit time distribution and found that while higher respiration rates induced by higher temperature increase the transit time because older carbon is respired, increases in productivity cause a decline in transit times because more young carbon is available to supply increased metabolism. The combined effect of increases in temperature and productivity results in a decrease in transit times, with the productivity effect dominating over the respiration effect. By using an ensemble of simulation trajectories from the Carbon Data Model Framework (CARDAMOM), we obtained time-dependent transit time distributions incorporating the twentieth century global change. In these simulations, transit time declined over the twentieth century, suggesting an increased productivity effect that augmented the amount of respired young carbon, but also increasing the release of old carbon from high latitudes. The transit time distribution of carbon becomes more asymmetric over time, with more carbon transiting faster through tropical and temperate regions, and older carbon being respired from high latitude regions.

This article is part of the Theo Murphy meeting issue ‘Radiocarbon in the Anthropocene’.

## 1. Introduction

The time carbon needs to transit through terrestrial ecosystems is an important indicator of the capacity of the terrestrial biosphere to take up carbon from the atmosphere and store it in the terrestrial surface for a relevant period of time [1]. This time is encapsulated in the concept of *transit time* of carbon [2,3], which measures the time carbon atoms spend in the terrestrial biosphere, from photosynthesis until respiration. Transit times can be expressed as probability distribution functions, which represent the relative proportion of carbon leaving the terrestrial biosphere over a continuous range of ages. For systems in equilibrium with constant input (photosynthesis) and output rates (respiration), the mean of the transit time distribution can be obtained as the ratio of the total stocks of carbon to the total input or output flux, but the Earth system is now far from equilibrium conditions, particularly since the beginning of the industrial revolution. Thus, a major uncertainty exists on whether the transit time of carbon in the terrestrial biosphere has changed as a consequence of global environmental change.

Previous studies suggest that the mean transit time of carbon in the terrestrial biosphere is only a few decades, with a decline over time since the beginning of the industrial revolution [4–7]. However, these previous studies usually compute a *mean residence time* or *turnover time* as the ratio of carbon stocks over fluxes, which have been shown to depart considerably from the mean transit time of carbon obtained from more recent approaches that explicitly account for the temporal dynamics of the age of specific pools [7–10]. Mean transit time, mean residence time and turnover time are the same for a carbon cycle in equilibrium where carbon stocks do not change over time and photosynthetic inputs are equal to respiration outputs [2,9]. We avoid here the *residence time* term because for systems out of equilibrium, it can be interpreted as the age of the carbon stocks or the age of the carbon in the output flux, while transit time more explicitly conveys the concept of the age of carbon in the output flux and can be characterized by an entire probability distribution and not just a mean value. So far no study has attempted to characterize historical changes in the probability distribution of transit times at the global scale.

Impulse response functions [3], and the theory of compartmental dynamical systems [11,12], suggest that the shape of the transit time distribution is a mixture of exponential distributions (a phase-type distribution) in which most carbon leaves the terrestrial biosphere very fast and very small proportions remain for very long times [3,13]. It is well known that the mean of this type of distribution is usually highly skewed by the presence of very large values, and the median better represents typical values.

The mean transit time of carbon in the terrestrial biosphere might be skewed by large transit times in pools such as boreal peatlands and tundra soils, where small proportions of carbon fixed by photosynthesis may take centuries to appear in the respiration flux [14]. By contrast, the respiration flux in tropical forests and grasslands, which dominate the global gross primary production (GPP) flux [15,16], might be composed of recently fixed carbon [17]. In such a case, the difference between the mean and the median transit time for the entire terrestrial biosphere might be large, with the median value providing a better indication of fast metabolic processes and the mean value better indicating time lags in carbon transfers and slow metabolism.

It is likely that transit times of carbon in terrestrial ecosystems have not remained constant since the beginning of the industrial revolution. Different lines of evidence suggest that GPP has increased during the industrial period, most likely as a combination of ${\text{CO}}_{2}$ and nitrogen fertilization, forest regrowth in the northern hemisphere (NH) and fire suppression, among other factors [18–21]. Temperatures have also increased globally, with related increases in ecosystem process rates that lead to increases in ecosystem respiration fluxes [22]. It is likely that these changes have resulted in decreases in the mean transit time of carbon [7,8,10], but it is unknown whether other quantiles of the transit time distribution have responded in a similar way.

Differences between the mean and the median transit time of carbon in the terrestrial biosphere can give important insights on global scale processes that affect not only the terrestrial carbon cycle but also the entire biosphere–atmosphere carbon exchange rate. Here, we will develop the theoretical and computational framework to obtain the median and mean transit times of carbon, and apply it to a simple carbon model and a land surface model driven by the twentieth century environmental change.

The main objectives of this article are as follows: (a) to obtain estimates of the mean, median and 95% quantile of the transit time distribution of carbon in the terrestrial biosphere, and evaluate how these metrics have changed during the industrial period; and (b) to use the difference between the mean and the median transit time of carbon to evaluate the hypothesis that carbon transit times are becoming more similar (homogeneous) across the globe. In particular, we are interested in evaluating whether transit times are becoming faster and more similar across all latitudinal regions, or whether there are different responses for particular regions that make the entire transit time distribution more asymmetric.

We used two different models for this purpose. First, we used a very simple model with no spatial representation of carbon dynamics to assess the separate and combined effects of increases in productivity and cycling rates on transit times. Then, we used a spatially explicit model informed by observations to evaluate trajectories of transit times for the period from 1920 to 2015. In the following, we describe the theoretical basis of our approach, describe the two involved models and discuss the results in the context of the two objectives previously mentioned.

## 2. Methods

### (a) Description of the approach

We assume here that carbon cycling in the terrestrial biosphere is well characterized by a particular type of dynamical systems called *compartmental systems* [23–25]. These systems of differential equations generalize mass-balanced models and therefore generalize element and carbon cycling models in ecosystems [25]. In their most general form, we can write carbon cycle models as follows:

2.1 $$\frac{\text{d}x}{\text{d}t}=\dot{x}(t)=u(x,t)+\text{B}(x,t)\cdot x,$$

where $x\in R^{n}$ is a vector of ecosystem carbon pool contents, $u(x,t)\in R^{n}$ is a time-dependent vector-valued function of carbon inputs to the system and $B(x,t)\in R^{n\times n}$ is a time-dependent compartmental matrix. The latter two terms can depend on the vector of states, in which case the compartmental system is considered nonlinear. In case the input vector and the compartmental matrix have fixed coefficients (no time dependencies), the system is considered autonomous, and non-autonomous otherwise. At a steady state, the autonomous linear system has the general solution $x^{*}=-B^{-1}\cdot u$.

Age and transit time density distributions for autonomous systems in equilibrium can be computed using the formulas provided in the study by Metzler & Sierra [11], while for non-autonomous systems, these distributions can be computed using the framework provided in Metzler *et al.* [12], which will be described briefly in the following section.

### (b) Forward and backward transit times

The transit time for systems in equilibrium quantifies the time it takes for carbon to traverse an ecosystem, from the time it enters until the time it leaves. For systems out of equilibrium, it is important to distinguish between the forward and the backward transit times [26]. The forward transit time can be defined as the time it takes for carbon entering the system at time t to traverse the system, and the backward transit time is the time that it took carbon leaving at time t to traverse the system. In other words, the forward transit time looks at the future dynamics of the system, while the backward transit time looks at its past. Here, we will concentrate on the dynamics of the backward transit time, which represents the age of carbon respired by ecosystems at any given time. Therefore, when we refer in this article to transit time, we consider only the backward transit time.

To obtain backward transit time distributions for systems out of equilibrium, we need to obtain first the age distributions for systems in equilibrium before the anthropogenic perturbation. The vector-valued probability density function (pdf) of age of carbon in different pools for systems at steady state can be computed by [11]

2.2 $$f^{0}(a)={\left({\text{X}}^{*}\right)}^{-1}\;e^{a\cdot \text{B}}\cdot \frac{u}{||u||},\;a\geq 0,$$

where X is the diagonal matrix of the steady-state stocks, $\;e^{a\cdot B}$ is the matrix exponential computed for each value of a and ||u|| is the sum of the carbon inputs to all pools at steady state. From this pdf, we can derive the pdf of the system age

2.3 $$f_{A}(a)=-{\text{1}}^{⊤}\cdot \text{B}\cdot \;e^{a\cdot \text{B}}\cdot \frac{x^{*}}{||x^{*}||},\;a\geq 0,$$

and the pdf of the transit time

2.4 $$f_{T}(\tau )=-{\text{1}}^{⊤}\cdot \text{B}\cdot \;e^{\tau \cdot \text{B}}\cdot \frac{u}{||u||},\;\tau \geq 0,$$

for systems at a steady state. Here, $1^{⊤}$ denotes the transpose of the n-dimensional vector containing ones and $||x^{*}||$ the sum of the stocks of all pools at a steady state.

Out of equilibrium conditions, based on the initial age pdf ($f^{0}$) at steady state, we can obtain the vector.

$$\text{Mass in the system at time }t\text{ with age }a=\left\{ \begin{array}{ll} \Phi (t,t-a)\;\tilde{u}(t-a), & a<t,\\ \Phi (t,0)\;f^{0}(a-t)||x^{*}||, & a\geq t, \end{array}\right.$$

where Φ is a state-transition matrix. We obtain Φ by taking advantage of an existing numerical solution x(t), which we plug in the original system, obtaining a new compartmental matrix $\tilde{B}(t):=B(x(t),t)$ and input vector $\tilde{u}:=u(x(t),t)$. Then, the new linear non-autonomous compartmental system

2.5 $$\begin{array}{rl} \dot{y}(t) & \;=\tilde{\text{B}}(t)y(t)+\tilde{u}(t),\;t>t_{0}\\ \mathrm{and}\;y(t_{0}) & \;=x^{0} \end{array}\}$$

has the same solution x given by

2.6 $$x(t)=\Phi (t,t_{0})\;x^{0}+\int_{t_{0}}^{t}\Phi (t,\tau )\tilde{u}(\tau )\;d\tau ,$$

where $x^{0}=x^{*}$ is the vector of initial (equilibrium) stock sizes before the anthropogenic perturbation. We obtain the state-transition matrix as the solution of the following differential equation:

2.7 $$\frac{d}{dt}\Phi (t,t_{0})=\tilde{\text{B}}(t)\Phi (t,t_{0}),\;t>t_{0},$$

with the initial condition that $\Phi (t_{0},t_{0})$ equals the n-dimensional identity matrix. The backward transit time distribution is then computed as the weighted contribution of mass in the respiration flux with a given age [12].

Once the state-transition matrix Φ is known, carbon dynamics for the entire simulation period are known as well as the dynamics for radiocarbon. We computed the radiocarbon content in all pools for all times as well as for the respiration flux in our simulations following the procedure described in the study by Metzler *et al.* [27]. The atmospheric radiocarbon curves of Graven *et al.* [28] were used to incorporate radiocarbon in transient simulation runs from 1851 to 2015. All computations were performed with the open-source Python package CompartmentalSystems, which we used here to perform all computations with the complex land surface model, and the SoilR package [29] that was used for radiocarbon computations with the simple model.

### (c) Additional diagnostic metrics

Age and transit time distributions are computed at the grid-cell level for land surface models; however, it is challenging to analyse entire distributions for all grid cells for all simulation time steps. Therefore, it is necessary to aggregate information and produce diagnostic metrics that can give insights about the dynamics of the entire system in a reduced space.

Global backward transit times for the entire terrestrial biosphere were computed by summing, at each time step, the masses of carbon in all grid cells corresponding to each age bin of the transit time distribution. The resulting global backward transit time distributions represent the age of the respired carbon at each time for the entire respiration flux of all terrestrial ecosystems. This global backward transit time distribution can be characterized by its corresponding mean $E_{b}(t)$, median $m_{b}(t)$ and quantiles $Q_{b}^{\alpha }(t)$, where the subscript b represents backward transit time and α is a percentile (i.e. $m_{b}(t)=Q_{b}^{0.5}(t)$).

To assess how the shape of this global backward transit time distribution changes over time, we define the ratio h(t) as a measure of homogeneity of transit times

2.8 $$h(t):=\frac{m_{b}(t)}{\ln(2)E_{b}(t)}.$$

The motivation for this ratio comes from the fact that for an exponential distribution the median is the product of ln⁡(2) and the expected value. If the transit time distribution would have a shape close to an exponential distribution, then h≈1 and the transit times would have the higher degree of homogeneity. To better understand the concept of homogeneity in transit times, recall that for a one-pool system in equilibrium, mean age and mean transit time are equal, and both are exponentially distributed [8,11]. Therefore, the ratio h(t) gives an indication of how different is the system from a homogeneous system with no differences in rates among pools. The ratio h(t) also gives an indication of how different are the mean and the median transit times. Because larger transit time values have a stronger influence on the mean than the median, decreases in h over time would indicate increases in the contribution of older carbon to the total respiration flux.

Another metric to assess how homogeneous is the transit time for the entire terrestrial biosphere system is the ratio of the mean backward transit time to the mean system age,

2.9 $$h^{\prime }(t):=\frac{E_{b}(t)}{E_{A}(t)}.$$

For a one-pool system, mean age and mean transit time are equal, and therefore, the ratio would be approximately 1 for a homogeneous system. As $h^{\prime }$ deviates from the value of 1, it would indicate a higher degree of heterogeneity in carbon cycle processes.

### (d) Description of the models and the model output

To explore changes in backward transit times during the twentieth century and disentangle the separate effects of increasing productivity and surface temperature, we used two different terrestrial carbon models. A simple five-box model with no spatial representation of ecosystems was used to study the separate and combined effects of increases in inputs and in cycling rates on backward transit times. Then, we used a more complex model running on a global grid, parameterized using a Bayesian approach informed by global scale observations, and producing predictions with posterior prediction uncertainties. These two models and their simulations are described in the following sections.

#### (i) A simple five-pool model

We used the simple terrestrial carbon model developed by Emanuel *et al.* [30] to obtain equilibrium age distributions for major biospheric pools and for the entire biosphere. The model represents five main compartments: non-woody tree parts $x_{1}$, woody tree parts $x_{2}$, ground vegetation $x_{3}$, detritus/decomposers $x_{4}$ and active soil carbon $x_{5}$. In addition to its simplicity and tractability, there are two advantages of using this model over others: (1) it provides reasonable values of carbon stocks and fluxes for a pre-industrial biosphere; and (2) its impulse response function and distributions of system age and transit time have been studied previously [3,11,30]. In addition, simulation results from this simple model provide intuitive insights that help to understand results from the more complex land surface model.

The model, expressed as a linear autonomous compartmental system, is given by

2.10 $$\begin{array}{rl} \dot{x} & \;=u+\text{B}x,\\ & \;=\left(\begin{array}{c} 77\\ 0\\ 36\\ 0\\ 0 \end{array}\right)+\left(\begin{array}{ccccc} -77/37 & \;0 & \;0 & \;0 & \;0\\ 31/37 & \;-31/452 & \;0 & \;0 & \;0\\ 0 & \;0 & \;-36/69 & \;0 & \;0\\ 21/37 & \;15/452 & \;12/69 & \;-48/81 & \;0\\ 0 & \;2/452 & \;6/69 & \;3/81 & \;-11/1121 \end{array}\right)\left(\begin{array}{c} x_{1}\\ x_{2}\\ x_{3}\\ x_{4}\\ x_{5} \end{array}\right), \end{array}$$

where mass of carbon is given in units of PgC and fluxes in units of PgC ${\text{yr}}^{-1}$. Total carbon inputs to the terrestrial biosphere (GPP) are 113 PgC ${\text{yr}}^{-1}$.

We also ran this model for the industrial period using functions to perturb carbon inputs due to increases in atmospheric ${\text{CO}}_{2}$ concentrations and to perturb cycling rates due to changes in temperature. The approach is similar to that presented by Rasmussen *et al.* [8], using the following time-dependent functions to represent atmospheric ${\text{CO}}_{2}$ concentration ($x_{a}$) and surface temperature ($T_{s}$) for the period $t_{0}=1850$ until $t_{\text{max}}=2020$, where

2.11 $$x_{a}(t)=\frac{t_{0}\exp\left(0.0305(t-t_{0})\right)}{t_{0}+\exp(0.0305(t-t_{0}))-1}+284,$$

and

2.12 $$T_{s}(t)=15+\frac{4.5}{\ln(2)}\ln\left(\frac{x_{a}(t)}{285}\right).$$

The equilibrium GPP value from the model of Emanuel *et al.* [30] (u in equation (2.10)) was modified as follows:

2.13 $$u(t)=\left(1+2.5\beta (t)\ln\left(\frac{x_{a}(t)}{285}\right)\right)u,$$

with

2.14 $$\beta (t)=\frac{3\rho x_{a}(t)\Gamma (t)}{\left(\rho x_{a}(t)-\Gamma (t))(\rho x_{a}(t)+2\Gamma (t)\right)},$$

ρ=0.65, and

2.15 $$\Gamma (t)=42.7+1.68(T_{s}(t)-25)+0.012{\left(T_{s}(t)-25\right)}^{2}.$$

Consequently, productivity (GPP) in the model is affected only by increases in atmospheric ${\text{CO}}_{2}$. Although other processes may have contributed to increase global ecosystem productivity during the twentieth century, we used this ${\text{CO}}_{2}$-driven approach for simplicity and comparability with the results from the study by Rasmussen *et al.* [8], and our transit time computations can be more broadly interpreted as a result of increases in productivity during the twentieth century.

Cycling rates, encoded in the matrix B of equation (2.10), were modified according to a rate-modifying function ξ(t) of the form

2.16 $$\xi (t)=2^{0.1T_{s}-1.5}.$$

To explore the separate and combined effects of increasing productivity and surface temperature on backward transit times during the industrial period, three separate simulations were run: (1) increasing ${\text{CO}}_{2}$ and constant temperature; (2) increasing temperature and constant ${\text{CO}}_{2}$; and (3) combined increases in temperature and ${\text{CO}}_{2}$.

#### (ii) CARDAMOM ensemble runs

To explore global scale patterns in transit times, we used the carbon data model framework (CARDAMOM), a model data-fusion system that combines observations of the carbon cycle and optimizes parameters of the DALEC ecosystem model [31,32]. In a global grid mesh ($4^{\circ }\times 5^{\circ }$ latitude, longitude), CARDAMOM estimates best values for initial conditions, ecosystem parameters and carbon pool histories at each grid cell [33,34]. The assimilated data include global maps of solar-induced fluorescence, net biosphere exchange, leaf area index, soil organic matter and biomass [32]. CARDAMOM produces ensembles of posterior parameters and prediction trajectories. Here, we used a sample of 50 prediction trajectories from which we reconstructed a compartmental dynamical system following the procedure described in the study by Metzler *et al.* [27]. This algorithm takes all the stocks and fluxes among pools predicted by the model, and reconstructs a state-transition operator $\Phi (t,t_{0})$ from which time-dependent transit times and radiocarbon dynamics can be computed following the procedure described earlier.

Simulation runs go from 1920 to 2015 with increasing ${\text{CO}}_{2}$ (with stomata response) and changing climate. Climate inputs (vapour pressure deficit, maximum and minimum daily temperatures, shortwave solar radiation and precipitation) for model runs were taken from monthly CRUNCEP v7 reanalysis [35]. Atmospheric ${\text{CO}}_{2}$ concentrations were taken from historical values of globally averaged annual mean from CMIP5 [36] together with values from RCP8.5 [37] for 2006–2015.

CARDAMOM simulates emission fluxes by fire based on burned area inputs and optimized emissions factors relating burned area to emission rates of CO and ${\text{CO}}_{2}$. We used the Global Fire Emissions Database (GFED) V4.1s burned area to drive CARDAMOM during the observational period (1997–2015) [38]. Before the observational period, we synthesized burned area at each point for the last century by randomly resampling from the distribution of observed GFED V4.1s observations for a given month.

## 3. Results

### (a) Productivity and respiration controls on transit times predicted by a simple model

**Figure 1.**
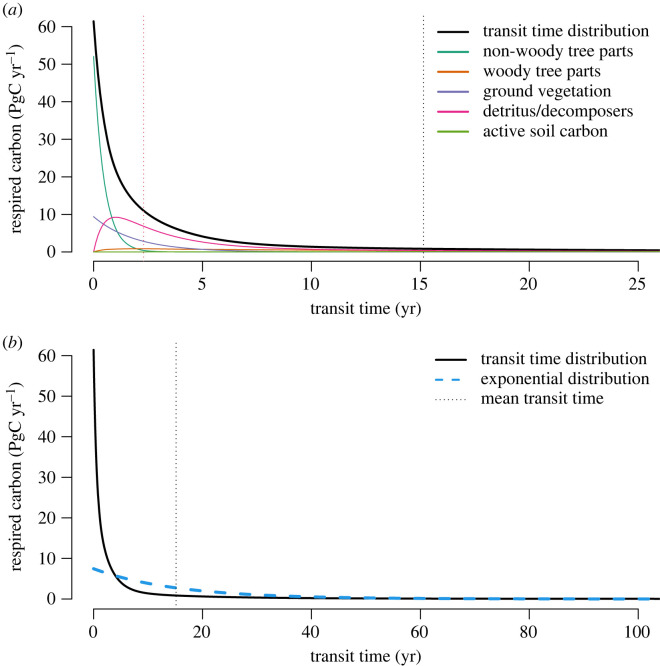
Transit time distribution of carbon entering the terrestrial biosphere as predicted by the model in [30]. The vertical axis represents the amount of carbon respired at any given year, in units of PgC ${\text{yr}}^{-1}$, that corresponds to the transit time in the horizontal axis. The area under the curves gives the total amount of respired carbon at any given year, in units of PgC. (a) Coloured lines represent the contribution of different pools to the transit time distribution. The vertical black line represents the mean transit time, 15.4 years, and the vertical red line represents the median transit time, 2.3 years. (b) Comparison between the transit time distribution and an exponential distribution with the same mean value (vertical line). Notice the difference in scale in the x-axis.

At equilibrium, the model by Emanuel *et al.* [30] predicts a total amount of GPP of 113 PgC ${\text{yr}}^{-1}$. From this amount, 50% of the carbon is returned back to the atmosphere in 2.3 years, and 95% in 74.5 years (figure 1). The mean transit time predicted by this model is 15.4 years with a standard deviation of 45.0 years, which suggests that the transit time distribution is far from an exponential distribution and has a long tail. This transit time distribution implies that most carbon fixed by photosynthesis in the terrestrial biosphere is respired very quickly within a few years, with only very small proportions staying in the terrestrial biosphere for longer timescales.

**Figure 2.**
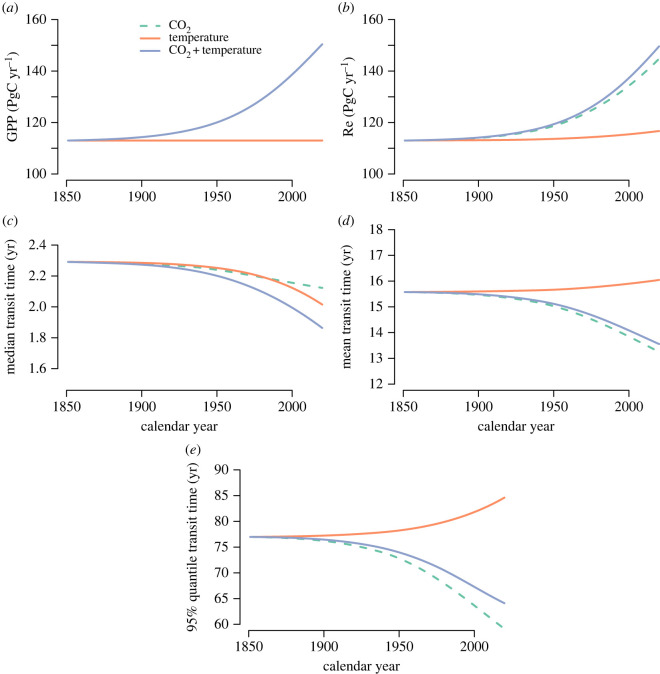
Predicted trends in (*a*) gross primary production (GPP), (*b*) ecosystem respiration (Re), (*c*) median transit time, (*d*) mean transit time and (*e*) 95% quantile of the transit time distribution as predicted from simulation experiments with the simple terrestrial carbon model of Emanuel *et al.* [30]. Line colours indicate simulations driven by increases in productivity induced by increases in atmospheric ${\mathrm{CO}}_{2}$ (dashed green), by an increase in temperature (orange) and by an increase in both ${\mathrm{CO}}_{2}$ and temperature (purple). In (a), lines for ${\mathrm{CO}}_{2}$ and ${\mathrm{CO}}_{2}$ + temperature overlap.

The simulation with increases in atmospheric ${\text{CO}}_{2}$ alone not only increased GPP but also increased Re (figure 2*a*). This higher amount of carbon inputs into the terrestrial biosphere increased all flows of carbon to the different compartments, with a subsequent increase in the flow of carbon out of the compartments. Therefore, more carbon of younger ages appeared in the respiration flux, decreasing the mean and the 95% quantile of the distribution of backward transit times (figure 2*b*), but without any significant effect on the median backward transit time. Compared with the values in 1850, the mean and the 95% quantile of the backward transit time distribution decreased in 2020 by 15 and 23%, respectively, while the median only decreased by 7%.

The increase in temperatures alone increased Re (figure 2*c*), but this effect was relatively small compared with the change in GPP induced by ${\text{CO}}_{2}$. The mean and the 95% quantile of the backward transit time had an increase due to a larger contribution of old soil carbon to the total respiration flux (figure 2*d*). Compared with the values in 1850, the mean and the 95% quantile of the backward transit time distribution in 2020 increased by 3 and 10%, respectively, while the median transit time showed a decrease of 12%. Because temperature changes increase the decomposition of both young and old carbon, the release of older carbon affected the increase in the mean and the 95% quantile of the transit time distribution, while the increased release of young carbon caused a decrease in the median transit time.

In the simulation with the increase in both ${\text{CO}}_{2}$ and temperature, both GPP and Re increased over time, with a larger respiration flux in this simulation in comparison with the simulation with ${\text{CO}}_{2}$ alone (figure 2*e*). The mean, median and 95% quantile of the backward transit time decreased in this simulation (figure 2), representing the dominant role of increased productivity on younger carbon being respired. Relative to 1850, the mean, median and 95% quantile of the backward transit time distribution decreased in 2020 by 13, 19 and 17%, respectively.

**Figure 3.**
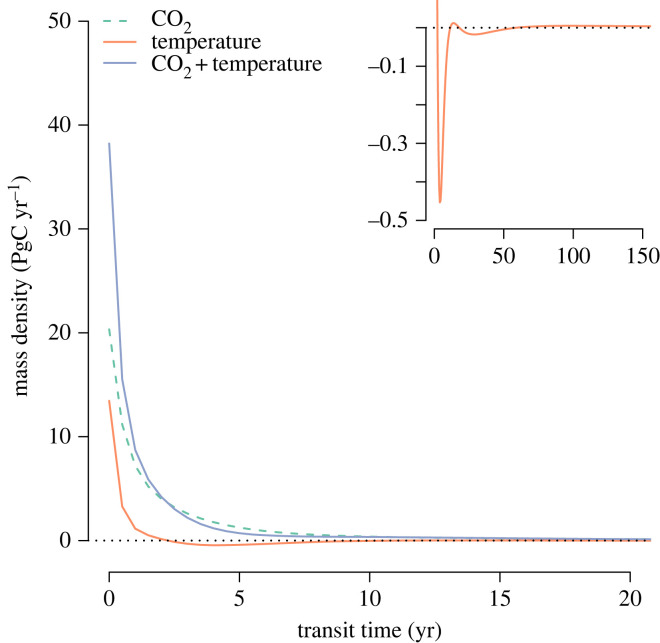
Differences between backward transit time distributions of the different simulations at year 2020 with respect to the equilibrium transit time distribution of the model of Emanuel *et al.* [30]. The inset on the upper right shows the results from the simulations of temperature change only at a different scale to highlight the differences in respiration at different ranges of transit times. Axes in the inset correspond to the same variables and units as the main figure with only differences in scale.

To better observe the effect of the different simulations on the entire transit time distribution, we subtracted the equilibrium distribution (figure 1) from the backward transit time distributions obtained at year 2020 (figure 3). This comparison shows that increases in ${\text{CO}}_{2}$ and temperature, alone and in combination, lead to an increase in the amount of respired carbon of young ages. However, the difference in the age of respired carbon between the equilibrium simulation and the temperature alone simulation showed a much more complex response. At fast transit times (0–2 years), there was higher respiration not only due to increased temperature at year 2020 but also with a decline at intermediate ages (2–11 and 18–59 years), and increases at transit times between 12 and 18 years, and from 59 years onwards (figure 3, inset). This complex response to increases in temperature suggests that the increase in process rates interacts with the size of the carbon stocks in the different pools, with some pools responding more strongly than others and thus creating these different age periods of increased and reduced responses. The small increase in respiration at transit times higher than 59 years is large enough to lead to an overall increase in $E_{b}(t)$, $m_{b}(t)$ and $Q_{b}^{0.95}(t)$ as observed in figure 2*d*.

**Figure 4.**
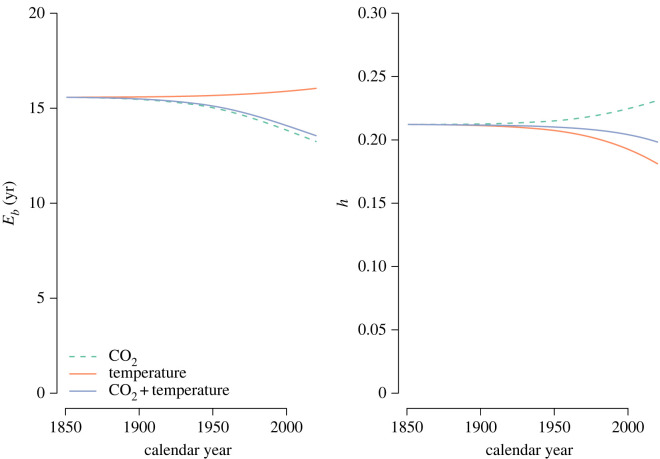
Mean backward transit time $E_{b}(t)$ and the h(t) ratio for the three separate simulations performed with the model presented by Emanuel *et al.* [30].

The ratio h(t) computed for the different simulations with the model of Emanuel *et al.* [30] showed that the entire backward transit time distribution is far from the value 1 that would indicate system homogeneity (figure 4). The values of h(t) were always below 0.24 for all simulations, which also indicates that the mean is at least six times as large as the median of the backward transit time distribution ($E_{b}>m_{b}/(\ln(2)0.24)=6.0m_{b}$). Interestingly, in the simulation with changes in ${\text{CO}}_{2}$ only, the difference between mean and median transit time decreased, very likely because the increase in inputs had a larger effect on the mean than on the median transit time (figure 4).

**Figure 5.**
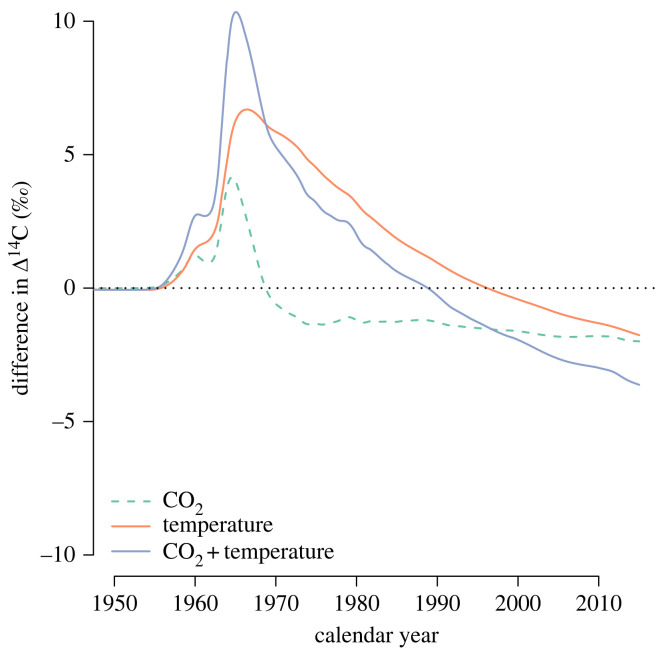
Radiocarbon in respired ${\mathrm{CO}}_{2}$ predicted by the model by Emanuel *et al.* [30] reported as the difference between the predictions from each simulation and a reference simulation with the carbon cycle at equilibrium. Positive values indicate a respiration flux with $\Delta^{14}C$ higher than the values of the model at equilibrium.

The dynamics of radiocarbon over the historical period showed contrasting trends for the three simulations (figure 5). To facilitate comparisons, we present in figure 5 the difference in radiocarbon, expressed as $\Delta^{14}\text{C}$, between a simulation with the model at equilibrium and the manipulations of temperature and ${\text{CO}}_{2}$. The results show that the increase in productivity produced a higher release of radiocarbon from the terrestrial biosphere shortly after the bomb spike caused mostly by the response of fast cycling pools such as ground vegetation, non-woody tree parts and detritus (figure 13). After the mid-1960s, faster transit times result in lower radiocarbon values in comparison with the model at equilibrium. For the temperature-only simulation, more radiocarbon is released after the bomb spike in comparison with the simulation at equilibrium, but the response is delayed by a few years, mostly because of the slow response of woody biomass and soil pools. After the 1990s, the radiocarbon released by the system is lower than for equilibrium conditions due to the contribution of slow cycling pools that release older radiocarbon than in the equilibrium simulation. The combined effect of temperature and ${\text{CO}}_{2}$ increase resulted in a relatively high and fast response of radiocarbon release after the bomb spike contributed by the fast cycling pools, with a subsequent rapid decline over the subsequent decades, indicating the contribution of the slower pools to the respiration flux (figure 13). Overall, radiocarbon appears to be sensitive to changes in the contribution of different carbon pools to the respiration flux and can serve to identify factors that may contribute to changes in backward transit times.

### (b) Historical simulation with CARDAMOM

We look now at an ensemble of historical simulations from CARDAMOM. Ensemble members show a consistent increase in GPP for the period 1920–2015, mostly explained by the ${\text{CO}}_{2}$ fertilization effect and regrowth from fires (figure 12). Respiration also increased consistently during this period for all ensemble members, mostly due to a combination of increases in GPP and temperature-induced respiration [32].

**Figure 6.**
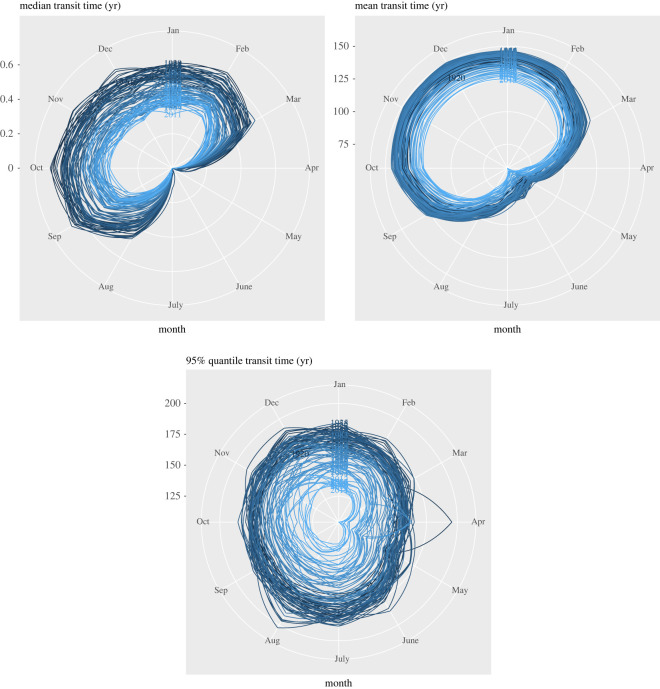
Seasonal patterns of the median, mean and 95% quantile of the backward transit time distribution of carbon in the terrestrial biosphere as predicted by CARDAMOM. The gradient from dark to light blue represents the transition in time from 1920 to 2015.

**Figure 7.**
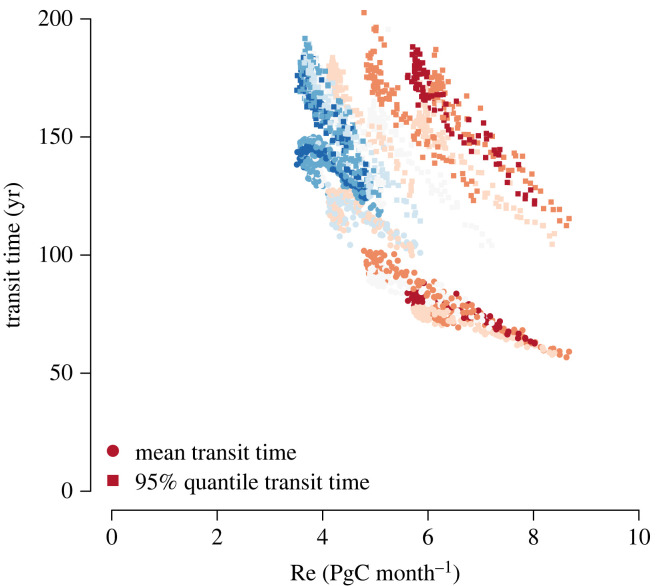
Relationship between ecosystem respiration Re and backward transit time (mean and 95% quantile of backward transit time distribution). Data points represent averages across all ensemble members, and colour gradient ranges from NH summer months (red) to NH winter months (blue).

Backward transit times showed a seasonal pattern that closely followed the pattern of ecosystem respiration in the NH. When respiration is high during the NH summer months, younger carbon appears in the respiration flux (figures 6 and 7). The simulations showed a consistent decrease in the amplitude of the seasonal pattern of backward transit times from 1920 to 2015, with consistently less older carbon respired throughout the years.

The median transit time showed a value of approximately 0 year for the month of June for the entire simulation period. Because in DALEC, the underlying model of CARDAMOM, autotrophic respiration is subtracted immediately after GPP fixation, the entire autotrophic respiration flux has an age of 0 year [39]. This implies that during the summer months of the NH, the respiration flux in the terrestrial biosphere is dominated by the autotrophic component with very small contributions from heterotrophic respiration. During the winter months, the contribution from NH autotrophic respiration decreases and the age of the respired carbon increases. Nevertheless, the median transit time in these simulation is never higher than 0.5 year and declined over the simulation period (figure 6).

**Figure 8.**
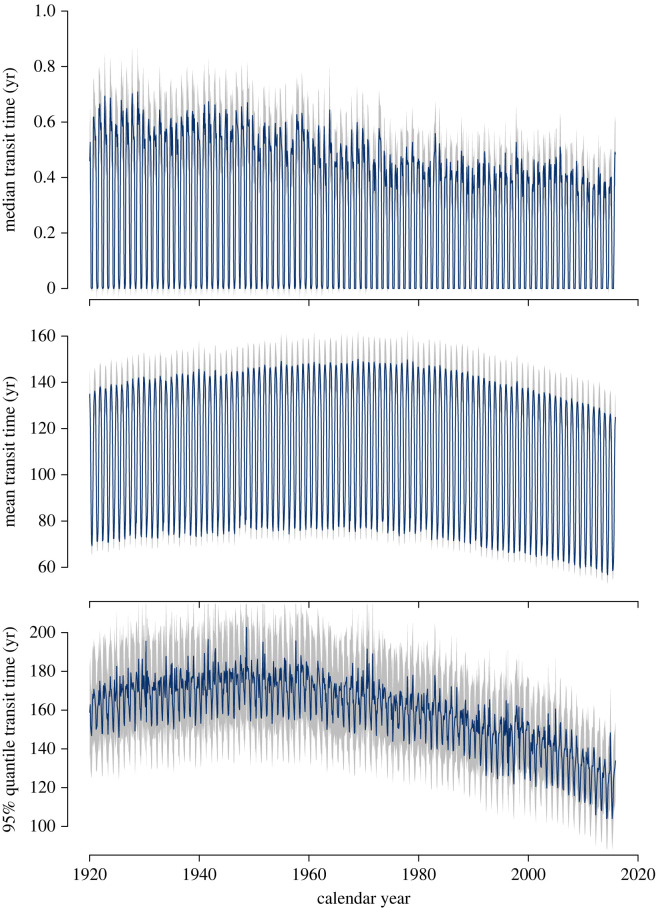
Median, mean and 95% quantile of the backward transit time of carbon predicted by CARDAMOM model runs. Coloured lines represent ensemble averages, and the background grey colour represents their standard deviation.

For the mean transit time, the seasonal variability is weaker than for the median transit time, but nevertheless a decline in the amplitude of the seasonal cycle is also observed for this variable. In the most recent years, the mean transit time varied in a range from 125 years in winter months to about 60 years in the summer months. Overall, an increasing trend in mean transit time was obtained from the 1920s to the 1980s and a subsequent decline afterward (figure 8).

The 95% quantile of the transit time distribution showed a strong decreasing trend from the 1960s to the 2010s (figure 8), and with less seasonal differences than the median and mean of the distribution (figure 6). This trend in the 95% quantile shows that less old carbon is being respired over the simulation period, contributing to the declining trend also observed for the mean transit time.

**Figure 9.**
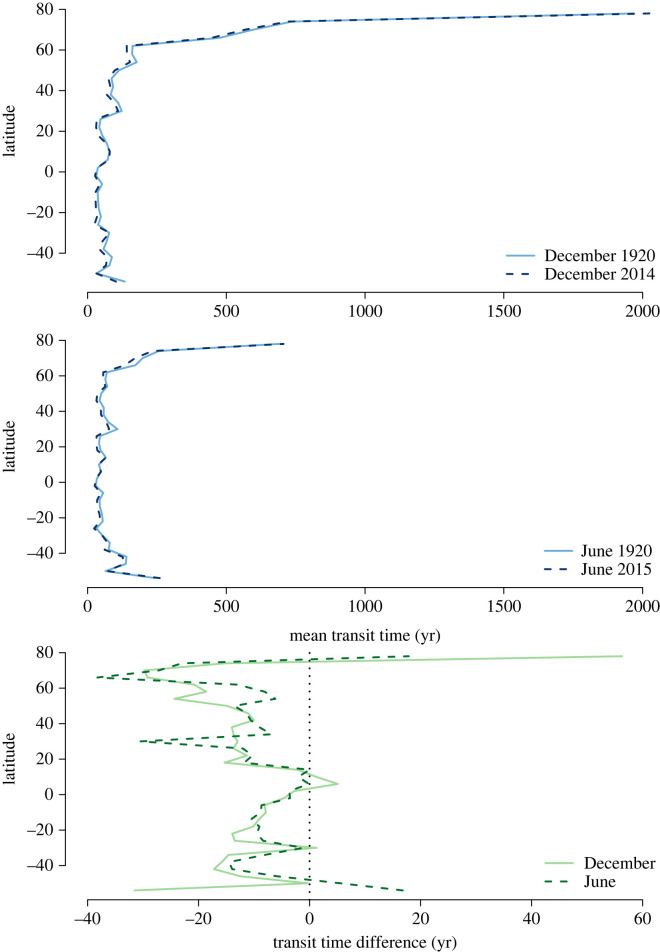
Latitudinal distribution of the mean backward transit time for NH winter and summer months in 1920 and 2014/2015. The upper panel represents the latitudinal average across all ensemble members for winter, and the middle panel for summer. The lower panels represents the difference in mean backward transit times between 1920 and 2014/2015.

The highest values of transit times were obtained for high latitude northern regions, where the mean transit time was as high as 2000 years during winter months. During summer months, the mean transit time decreases considerably in northern high latitude regions and is not larger than 800 years (figure 9). In most of the temperate and tropical regions, the mean transit time is lower than 100 years with small differences between summer and winter months.

The difference in mean transit time between the year 1920 and 2015 showed contrasting patterns across latitudes. In high latitude northern regions, respired carbon was older in 2015 than in 1920. However, for most temperate and tropical latitudes, respired carbon was younger in 2015 than in 1920. This latitudinal difference suggests a dominant role of old soil carbon in arctic regions, and a more dominant role of younger carbon in temperate and tropical regions.

**Figure 10.**
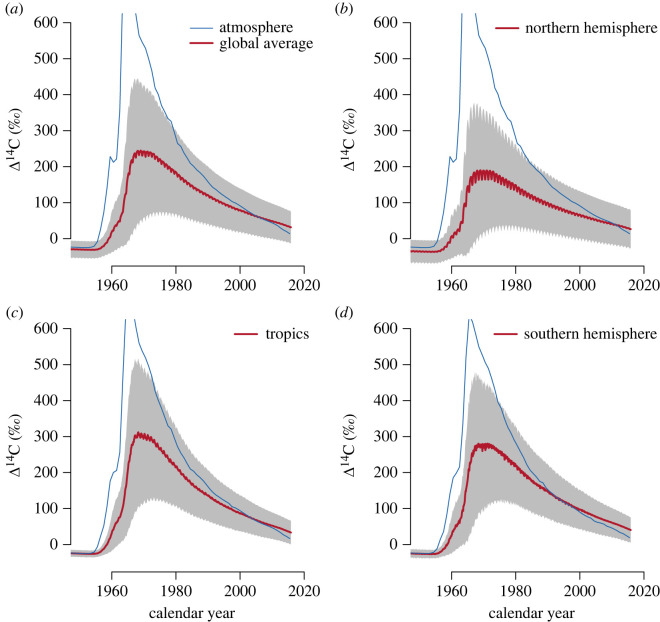
Average values of radiocarbon in respired ${\mathrm{CO}}_{2}$ from terrestrial ecosystems (red lines) as predicted by the CARDAMOM ensemble runs. (*a*) Average across all grid cells and ensemble members (red line) ± standard deviation (shaded area). (*b*) Average for grid cells between 34 and $78^{\circ }$ N across all ensemble members. (*c*) Average for grid cells between −30 and $30^{\circ }$ N across all ensemble members. (*d*) Average for grid cells between −50 and $-34^{\circ }$ N across all ensemble members. Atmospheric radiocarbon in the atmosphere for the different hemispheric zones from Graven *et al.* [28] is provided as a reference (blue lines).

Latitudinal differences in transit time were also well expressed in terms of differences in the radiocarbon signature of the respired ${\text{CO}}_{2}$ (figure 10). Aggregating grid cells in latitudinal bands for southern hemisphere (−58 to $-22^{\circ }$ N), tropics (−22 to $22^{\circ }$ N) and NH (22 to $78^{\circ }$ N), we see that the incorporation and release of bomb radiocarbon was consistent with the patterns observed for transit times. In tropical and southern hemisphere latitudes, the incorporation and release of radiocarbon is faster than in the NH where old radiocarbon diluted the respiration signal. A stronger seasonality in respired radiocarbon was also obtained for the temperate latitudes, consistent with the patterns observed for transit time.

**Figure 11.**
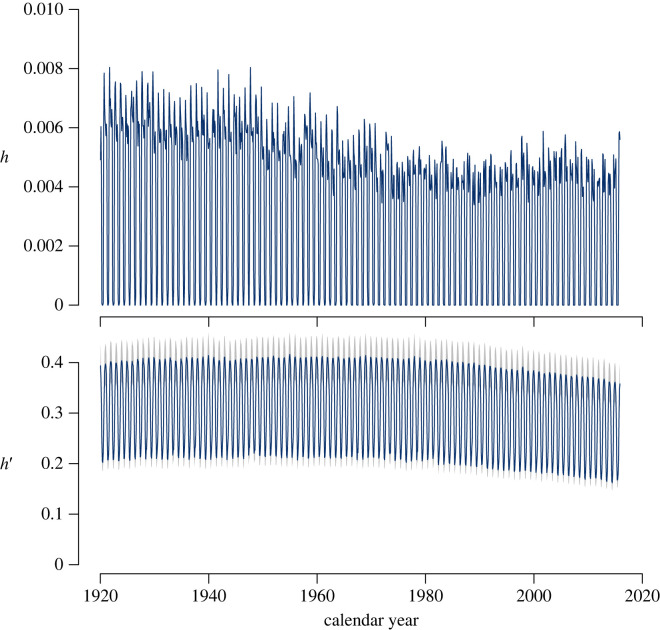
Homogenization ratios h(t) and $h^{\prime }(t)$ from CARDAMOM model runs. Colour lines represent ensemble averages, and the background grey colour represents their standard deviation.

**Figure 12.**
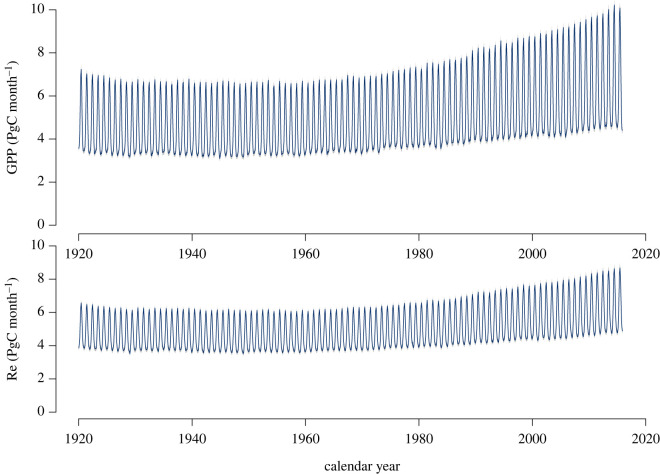
Gross primary production (GPP) and ecosystem respiration (Re) predicted by CARDAMOM. Values are averages across all ensemble members.

**Figure 13.**
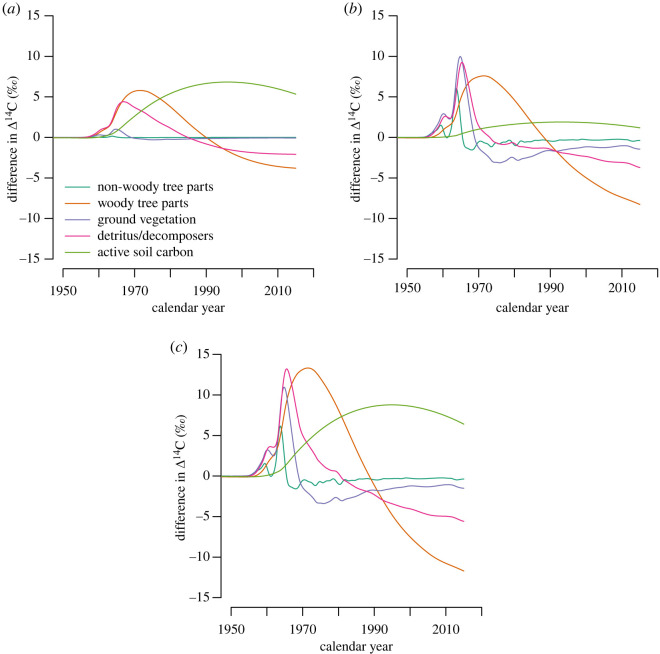
Differences in $\Delta^{14}C$ for individual pools between a simulation with the model of Emanuel *et al.* [30] at equilibrium and the different simulation experiments. (*a*) Difference with the simulation of temperature increase only. (*b*) Difference with the simulation of ${\mathrm{CO}}_{2}$ increase only. (*c*) Difference with the simulation of temperature and ${\mathrm{CO}}_{2}$ increase in combination.

The homogenization ratios h(t) and $h^{\prime }(t)$ computed for the CARDAMOM ensemble showed a slight decline over time, going further away from the value of 1 (figure 11). These results indicate that the difference between the mean and the median backward transit times increased during the simulation period for all ensemble members. It also indicates that the difference between the mean age of carbon and the mean backward transit time increased during the simulation period. These ratios capture the different responses of transit times across latitudes and carbon pools, and suggest that respired carbon from the terrestrial biosphere is becoming more heterogeneous, with more and older carbon being respired from higher northern latitudes, and more younger carbon respired from temperate and tropical regions.

## 4. Discussion

Simulations with two different terrestrial carbon cycle models with contrasting levels of complexity suggest that the transit time of carbon through the terrestrial biosphere may have been declining since the twentieth century. This decrease in the age of respired carbon could be explained by two main factors, increases in global scale GPP and temperature-driven increases of cycling rates in carbon compartments. In the two models analysed, increases in gross primary production increased the amount of carbon transfer to the network of ecosystem compartments and therefore increased respiration. As a result, more carbon of younger ages was respired due to this increase in productivity.

The response to temperature-induced cycling rates may be more complex than what could be expected by simple temperature response functions such as Arrhenius or Q10 [40–42]. Although increases in cycling rates in fast cycling compartments such as foliage and litter promotes respiration of younger carbon, increases in cycling rates also increase the decomposition of older carbon in slow cycling pools such as coarse woody debris and soils [14,43]. The net result in transit times may depend on the proportional contribution of young and old carbon pools to the total respiration flux. The CARDAMOM simulations predict an overall increase in the age of respired carbon during the earlier part of the twentieth century, particularly at high latitudes, but proportionally more young carbon appeared in the respired flux after the 1980s. High latitude regions respired older carbon in the earlier part of the twenty-first century in comparison with the beginning of the twentieth century, possibly due to the large stocks of old carbon still available for decomposition in this region.

Fires may also play an important role in the overall trend of backward transit times computed here with the CARDAMOM ensemble. Although we were not able to separate the fire effect explicitly in our analysis, increases in fire frequency and severity may have promoted an overall increase in old carbon returned to the atmosphere in the earlier part of the twentieth century, and a later increase in young carbon in regions with subsequent fire events.

Our analysis is the first to present quantiles of the transit time distribution for the entire terrestrial biosphere. The median and the 95% quantiles of this distribution showed relatively different behaviours in comparison with the mean transit time. The median transit time gives an indication of fast metabolic processes and how they vary seasonally, while the 95% quantile of the transit time distribution indicates dynamics of older carbon that change at different timescales than the dynamics of the active metabolic carbon. The median transit time declined consistently during the entire simulation period, while the mean and 95% quantile only started to show a decline during the later part of the twentieth century. These different behaviours indicate that carbon metabolism may respond very differently to global change drivers according to how much and how old is the carbon in different pools in different ecosystems.

The decrease in median transit time and increase in mean and 95% quantile for some regions suggest that the entire transit time distribution is becoming more asymmetric. Younger carbon respired from tropical and temperate regions moves the 50% quantile of the distribution towards the left, while increases in older respired carbon from high northern latitude regions move the right tail of the distribution further to the right. We were able to assess this change in asymmetry of the transit time distribution with the ratios of median to mean transit time and mean transit time to mean age. Although the response of these ratios was stronger for the simple model than for the more complex model that includes more processes, we believe that this change in asymmetry may be an important characteristic of the contemporary terrestrial carbon cycle that has not been explored in detail yet and deserves further study. The terrestrial biosphere may be moving to a state in which there are more dissimilarities with regard to transit times across ecosystems, with less predictability about the future transit of carbon through the terrestrial biosphere.

Nevertheless, our estimates of backward transit time with the CARDAMOM ensemble may have significant underestimations. As mentioned earlier, DALEC assumes that autotrophic respiration occurs immediately after respiration [31,34], and therefore, the age of respired carbon from autotrophic pools is zero [39]. In reality, carbon in autotrophic pools such as foliage, stem and roots may take between a few hours to a decade to appear in the respiration flux [44–47]. Therefore, the median backward transit time computed here may be underestimated by about 10 years.

The predictions presented in this study are based on models and difficult to corroborate with observational and experimental data. However, we consider these results to provide insights that may promote future studies. Radiocarbon measurements in carbon pools and in respired ${\text{CO}}_{2}$, in combination with ecosystem models, may help to better quantify the transit time of carbon in terrestrial ecosystems. For instance, Trumbore & De Camargo [48] used a number of radiocarbon measurements in ecosystem pools to estimate a mean transit time of about 3–7 years for central Amazon forests. This estimate of transit time is not very far from the estimate of about 11 years obtained from a model-data assimilation study in a tropical forest in Colombia [17]. For a temperate forest in North America, Phillips *et al.* [49] estimated a mean transit time that ranged between 1 and 19 years using measurements of ${\;}^{14}{\text{CO}}_{2}$ below the forest canopy. These are some of the first studies to obtain an estimate of the mean transit time of carbon in forest ecosystems, but they should be expanded to capture seasonal and inter-annual dynamics. According to the model predictions from this study, the mean transit time from tropical and temperate forests should be declining, and long-term monitoring of radiocarbon in terrestrial ecosystems may help to corroborate these model predictions. Our simulation results showed that radiocarbon in respired ${\text{CO}}_{2}$ may be declining fast for most of the tropics and temperate regions, and therefore, repeated measurements of radiocarbon in ${\text{CO}}_{2}$ below forest canopies may help to relate changes in this variable to changes in transit times.

A much larger challenge would be to corroborate with observations global scale trends in transit times, not just trends for particular ecosystems. For this purpose, tropospheric ${\;}^{14}{\text{CO}}_{2}$ measurements may be of outmost help. Atmospheric radiocarbon is currently declining at a rate between −5 and $-4\text{‰}\;{\text{yr}}^{-1}$ due to the combustion of fossil fuels [50,51]. Radiocarbon from the bomb period, still present in the terrestrial biosphere, may add a significant level of seasonal variability that could be used to detect seasonal and inter-annual patterns in biosphere transit times [51,52].

## 5. Conclusion

Simulations from two contrasting terrestrial carbon models suggest that the mean transit time of carbon in the terrestrial biosphere may be declining, but with important differences among the quantiles of the transit time distribution for different latitudinal regions. While in temperate and tropical forests the age of respired carbon may be declining as a combination of increased productivity and higher temperature, the age of respired carbon in high latitude regions may be increasing due to respiration of century-old carbon. These different dynamics are likely increasing the asymmetry of the transit time distribution for the entire terrestrial biosphere, with more young carbon skewing the distribution to its left part and more older carbon skewing the distribution to its right part.

As a consequence, additional carbon from increased productivity may be spending less time in terrestrial ecosystems and more older carbon is being destabilized and emitted to the atmosphere. These changes may have important implications for global climate, with a terrestrial carbon cycle becoming more active and storing carbon for less time as indicated by the decreasing median transit time, and at the same time respiring older and older carbon reserves as indicated by the decreasing homogeneity ratios.

## Data Availability

This article has no additional data.

## Appendix A. Supplementary figures

See figures 12 and 13.
